# Effects of vibrotactile-enhanced music-based intervention on sensorimotor control capacity in the hand of an aging brain: a pilot feasibility randomized crossover trial

**DOI:** 10.1186/s12877-021-02604-0

**Published:** 2021-11-23

**Authors:** Hsiu-Yun Hsu, Che-Wei Lin, Yu-Ching Lin, Po-Ting Wu, Hirokazu Kato, Fong-Chin Su, Li-Chieh Kuo

**Affiliations:** 1 Department of Physical Medicine and Rehabilitation, National Cheng Kung University Hospital, College of Medicine, National Cheng Kung University, Tainan, Taiwan; 2 Department of Occupational Therapy, College of Medicine, National Cheng Kung University, Tainan, Taiwan; 3 Medical Device Innovation Center, National Cheng Kung University, Tainan, Taiwan; 4 Department of Biomedical Engineering, College of Engineering, National Cheng Kung University, Tainan, Taiwan; 5 Department of Physical Medicine and Rehabilitation, College of Medicine, National Cheng Kung University, Tainan, Taiwan; 6 Department of Orthopedics, College of Medicine, National Cheng Kung University, Tainan, Taiwan; 7 Graduate School of Information Science, Nara Institute of Science and Technology, Ikoma, Japan

**Keywords:** Music-based intervention, Haptic, Sensorimotor, Hand function

## Abstract

**Background:**

Music-based interventions (MBI), using music as a therapeutic medium, has been utilized as a promising strategy for motor relearning and shaping. However, currently, MBI with active performance training is restricted to being extensively applied for patients with various levels of defects in fine motor skills and cognitive functions. Therefore, the integration of vibrotactile stimulation with MBI has been adopted as a motor training strategy intended to enhance motor learning through use of vibration stimuli. The current study was designed to investigate differences in the sensorimotor performance of older adults’ hands under baseline, a single session of active MBI, and vibrotactile-enriched MBI conditions.

**Methods:**

Thirty healthy older adults were recruited and randomized to receive either the single session of 30-min of vibrotactile-enriched MBI or 30-min of active MBI at the beginning of the experiment. After a one-week washout period, they switched their treatment programs and then were assessed to study the training effects of both approaches through measuring precision pinch performance, hand function, and sensory status.

**Results:**

The results of the Pinch-Holding-Up Activity test revealed a statistically significant difference in the FR_peak_ parameter (F = 14.37, *p* < 0.001, η^2^_p_ = 0.507) under the vibrotactile-enriched MBI condition compared to the baseline and active MBI conditions. In addition, significant beneficial effects were found on the results of the barognosis (F = 19.126, *p* < 0.001, η^2^_p_ = 0. 577) and roughness differentiation subtests (F = 15.036, *p* < 0.001, η^2^_p_ = 0.518) in the Manual Tactile Test for the participants in the vibrotactile-enriched MBI group. In addition, the participants under both the active MBI and vibrotactile-enriched MBI conditions exhibited better performance in the three subtests of the Purdue Pegboard Test as compared to under the baseline condition (*p* < 0.016).

**Conclusions:**

The findings indicated that vibrotactile-enriched MBI potentially improves the precision pinch performance of hands in healthy older adults. In addition, the add-on effect of vibrotactile stimulation to the MBI condition provides beneficial effects on the sensory functions of the upper extremities.

**Trial registration:**

NCT04802564.

Date of registration: 15/03/2021.

The first posted date: 17/03/2021.

## Background

The number and percentage of the older population is rapidly increasing worldwide. Aging declines skill performance related to physiological changes in skeletal muscles [1] as well as reduced functional integration of the sensory-motor system [2]. It is worth noting that manual dexterity of the hand has long been known as an early indicator of age-related functional decline [3]. A previous study revealed that aging strongly impacts motor performance such as fingertip force modulation requiring online sensory input [4]. Specifically, the population with old age might usually be reported with their impairment of motor adjustments in response to the environmental perturbation and task demands [5] which might impact on the execution of daily living activities. Thus, activities, such as music for maintaining and even promoting the sensorimotor control capabilities of an aging brain [6] should be given attention.

Sensory-augmented therapy has been proposed as a helpful adjunctive strategy that can be used to enhance the effects of motor retraining when integrated with conventional rehabilitation programs for patients with sensorimotor deficits [7, 8]. Vibrotactile sensation caused by a mechanical stimulus characterized by an oscillating motion has been reported to enhance muscular function as well as neuronal activity [9]. A recent study revealed that the activity of the primary sensorimotor area is increased significantly during processing of high-frequency vibrotactile information [10]. In addition, light touch sensation has been shown to be improved in patients’ hands when performing a specific task with vibrotactile stimuli on the dorsum of the wrist [11]. This effect may be due to the enhancement of the excitability of sensory neurons through interneuronal connections during task execution [12].

In addition to vibrotactile stimuli, sensory-based interventions to improve hand function also include visual and auditory strategies. Music-based intervention (MBI), using music as a therapeutic medium, has been utilized as a promising strategy of neuro-rehabilitation in the last decade [13]. The delivery mode of MBI includes the following main types: active, receptive and combined active and receptive intervention [14]. The information providing by music auditory stimuli induces plastic changes in the brain through the effects of auditory-motor entrainment and sensorimotor synchronization for patients with neurological impairment [15, 16]. The active mode of MBI has been reported to be effective in restoration of motor skills of upper limb [17]. Playing keyboards and drums have been reported as the most popular training regimes used to improve fine motor and gross motor coordination, respectively [18]. MBI with active performance is currently considered to be a practical framework based on neuroscience used for motor relearning and shaping, audio-motor coupling, and evoking emotional effects since individuals receive auditory melody and rhythmic feedback [19]. Recent evidence revealed that the finger dexterity of subacute stroke patients was improved through providing diverse sensory rewards for sequential finger movements based on keyboard playing programs [20]. Specifically, MBI with active performance increased the activity and connectivity between the auditory and motor cortical regions by providing individuals with auditory feedback for errors and real-time movement adjustments, which consequently boosted motor recovery of the upper limbs of stroke patients [21]. In addition to patients with neurological deficits, a recent study also revealed that active music therapy has effects on improving upper limb muscle power in community dwelling older adults [22]. However, the effects of MBI with active performance on the sensorimotor control capacity of an aging hand closely related to lifestyle and living quality are as yet not well known.

MBI with active performance mainly focuses on movement relearning and shaping through training in the use of musical instruments or specifically-designed electronic devices. Despite the fact that positive findings have been reported [23, 24], the current active MBI protocol has limitations related to widespread application in patients with different or varied levels of fine motor skills and cognitive functions. Therefore, musical haptics, a new concept of adopting vibrotactile stimulation in MBI, which convey the music information through skin of the finger pulps is suitable to enrich the musical listening experience [25] and improve significantly on sensorimotor function [26]. In addition, vibrotactile stimulation has been reported to enhance the performance of the affected arm in stabilization and reaching tasks given to patients suffering from neurological diseases [27]. However, the difference in the training effects between the active MBI and enriched MBI with vibrotactile stimulation on hand functions has not been appropriately investigated. Thus, the purpose of this study was to analyze the difference in the effects on sensorimotor control capacity in the hand of an aging brain across baseline, active MBI, and vibrotactile-enriched MBI conditions. For this purpose, a vibrotactile-enriched interfaces for MBI system was designed to assist with performing MBI in this study. We hypothesized that both one-session of active MBI, and vibrotactile-enriched MBI treatment paradigm may lead to improvements in sensorimotor performance of a hand. The significance of this study was a vibrotactile-enriched MBI system providing multi-sensory experience has been developed and the feasibility of clinical practice has been tested. The findings of this study would highlight the benefit of active and vibrotactile-enriched MBI on sensorimotor performance of a hand.

## Methods

### Study design

An assessor blinded, randomized controlled, crossover design was used in the study. The participants were randomly assigned in a 1:1 ratio into either a one-session active MBI group or a vibrotactile-enriched MBI group. After a one-week washout period, they switched treatment programs. The assessments were conducted with the time of pre-treatment as the baseline (T_b_), immediately followed by one-session of the active MBI (T_a_) and vibrotactile-enriched MBI (T_v_). Two assessors who underwent in-person training on assessments performing were blinded to the participant’s condition. One was an occupational therapist (for the Semmes-Weinstein monofilament and Purdue Pegboard Test measures) and the other one was a technician (for the pinch-holding-up activity and manual tactile test measure). The data were collected at the department of physical medicine and rehabilitation in a medical center setting in southern Taiwan.

### Participants

Thirty community dwelling healthy older adults were recruited based on an estimation of the effects obtained regarding hand performance using a previous sensory augmented rehabilitation program estimated with a 2-tailed alpha of 0.05 and a power of 0.95 [28]. The inclusion criteria for all the participants in this research group were as follows: (1) right-handed; (2) age ranging from 55 to 85; (3) no history of neurological or psychiatric illness, no severe vision or hearing loss, no abnormalities in the upper extremities, (4) the capacity to perform and maintain a pinch task with the thumb and index finger while lifting an object, (5) no previous musical instrument education, and (6) a score of 24 or more on the Mini Mental State Examination. Participants with difficulty following instructions, diagnosed with neuro-musculoskeletal disorders, or having a poor attention span were excluded. Prior to participation, each participant was asked to sign a consent form after being informed of the objectives and the related research procedures. Fifteen male and 15 female older adults between the ages of 55 and 85 (65.7 ± 5.6 years old) were recruited in this study. The participants were with an average educational level of 11.8 ± 3.2 years. All participants completed the required outcomes measurements for the three conditions.

### Randomization and allocation concealment

The participants were randomly allocated to either active MBI or vibrotactile-enriched MBI condition first by a computer-generated random number sealed in opaque envelopes. The therapist opened the envelope and found the treatment to be conducted in this participant.

### Equipment

**Fig. 1:**
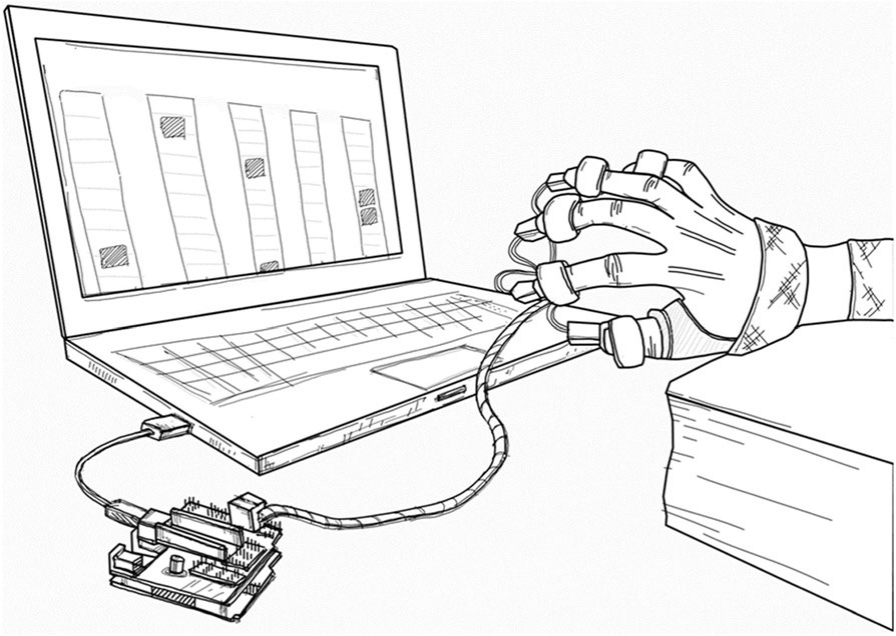
Illustration of the vibrotactile-enriched music-based intervention system

The vibrotactile-enriched interfaces for MBI system (Fig. 1) is a custom-made training apparatus composed of three distinct parts: (1) A laptop computer: This computer produces music output integrated with visual information in the form of a color bar moving on the screen display corresponding with the rhythmic elements of the music. (2) Haptic feedback component: To establish the haptic interface, an Arduino UNO microcontroller board was used as a microprocessor, which controlled and worked with five coin-shaped micro vibration motors (Model #1027, TAIWAINIOT™, Taiwan) to achieve the function of providing vibrotactile feedback in the system. The motor was driven at a voltage of 5 V_DC (direct current)_ with sinusoidal vibration applied to the fingertip at a frequency of approximately 200 Hz. The pulp of each digit was securely positioned in a specially designed 3D printing base as a reinforced structure with Velcro-fastenings. (3) Image classifier: The artificial intelligence deep residual network (ResNet50) was used for precise image recognition and the integration of the visual information from the moving color bar and vibrotactile stimuli in a timely manner.

### Intervention

Two training regimes, the active MBI and vibrotactile-enriched MBI regimes, based on a crossover study design with a one-week washout phase were applied for all the participants. The details of intervention delivering in this study was based on a reporting guideline of music-based interventions [29]. Ten familiar musical compositions with the tempo ranging between 49 and 100 beats/minute from the Beatles, consisting simple melody with periodic cycles, were pre-selected by the investigator as the materials for both the active MBI and vibrotactile-enriched MBI which provided participants with melodies and rhythms. Music was delivered by speakers with a volume controlled lower then 65 dB by an occupational therapist. The active MBI is aimed toward improving the participants’ fine motor skills through a 30-min “active practice” session. The participants were instructed to play music by pressing the keyboard with the corresponding digits guided by auditory and visual feedback displayed as a moving color-bar on the computer screen. The hand was allowed to perform different kinds of pressing activities, such as single and multiple digits pressing in a specific rhythm and melody on a computer keyboard. The difficulty level could be adjusted for each participant to meet the motor skill level. In the vibrotactile-enriched MBI, the system provided 30-min of vibrotactile stimuli synchronized with audio and visual information to each digit via small, coin-shaped vibration motors fitted on the pulps of the digits while the music was played. Different from the active MBI, the participants receiving the vibrotactile-enriched MBI were asked to receive multiple sensory information, including visual, auditory, and vibrotactile stimuli, but did not actively perform piano key pressing tasks. The stimulus sequences were same in both the active and vibrotactile-enriched MBI conditions.

### Outcome measures

#### Primary outcome measures

The pinch-holding-up activity (PHUA) test is a task-based assessment with high test-retest reliability (intra-class correlation coefficient ranged from 0.84 to 0.96) [30] was used for detecting the sensorimotor control of a hand with functional perspective. A 480 g-weight pinch apparatus with two load cells and one accelerometer used to detect the pinch force exertion of a hand and acceleration of the apparatus in space, respectively, were used to examine the pinch and load force coupling related to lifting performance of the upper extremity. The testing procedures comprised two phases, a holding phase and a lifting phase. The participants were instructed to pinch and lift the apparatus to a 5 cm height above the table and then asked to hold the device in this position for 5 s (holding phase). Afterwards, the pinch-apparatus was lifted from 5 cm to the height of 30 cm (lifting phase) above the table and then slowly lowered to the initial position. The total duration of data collection was approximately 15 s, where the maximum upward acceleration of the apparatus and peak pinch force exerted by the digits during the lifting phase of the test were recorded. Test procedures were repeated three times with between-trial resting interval of 1 minute. The peak pinch force during the lifting phase was defined as FP_peak_, and the maximum load of the object was defined as FL_max_. FR_peak_, the ratio between FP_peak_ and FL_max_, indicated the ability of a hand to adjust to the pinch force related to changes in the inertial load of the lifted object. The FR_peak_ for young healthy adult ranged between 1.77 and 1.98 [30]. FR_peak_ is a sensitive parameter used to assess the sensorimotor control of the hand in the form of a dynamic coordination model [31]. An elevated FR_peak_ indicated insufficient ability of sensorimotor control in a hand [32].

#### Secondary outcome measures

The manual tactile test (MTT) is a timed evaluation tool used to determine the discriminative sensation involved in active exploration of a hand with reported reliability, accuracy, and validity [33]. Three subtests in the MTT, barognosis, roughness differentiation, and stereognosis, were conducted for evaluating the hand perceptions of a participant related to distinguishing object characteristics - weight, roughness, and shape, respectively, with active touch while blindfolded. The test procedures for each subtest were repeated three times for each hand. The average time required to perform each test was calculated to arrive at the final score of that test. The lower the score obtained, the inferior of his discriminating sensibility.

The Semmes-Weinstein monofilament (SWM) test is the most responsive touch-pressure sensory test [34]. Higher score of the SWM test represented the poorer tactile sensation. It also has reliability and specificity for identifying a loss of protective sensation in seniors [35]. A full set of SWM contains 20 monofilaments, and each monofilament is labeled with a numerical marking, which is a log to the base ten of force in tenths of a milligram. When conducting the SWM test, the filaments are applied perpendicular to the pulp of each digit with a constant force onto the skin area for 1–1.5 s.

The Purdue Pegboard Test (PPT) is a test of the uni-manual and bimanual dexterity of the hands. It has been demonstrated to have high testing validity and reliability [36]. The test was carried out using a procedure in which pins are inserted into small holes with the dominant hand and both hands simultaneously in 30 s, as well as an assembly task that was performed for 60 s. Higher score is indicative of better finger dexterity.

### Statistical analysis

SPSS 19.0 for Windows (Statistical Package for Social Sciences Inc. Chicago, IL, USA) was used for the statistical analyses. The descriptive statistics were used to describe the means and standard deviations of the characteristics data and outcome measurements, including the results of the PHUA, MTT, SWM, and PPT. Normality in the data distribution was examined with a Shapiro-Wilk’s test (*p* ≥ 0.05). All variables fulfilled the normality criteria; therefore, the repeated measure ANOVA was used to determine if there are difference across the following conditions, the baseline, one-session of the active MBI, and one-session of vibrotactile-enriched MBI. Statistical significance was set at *p* < 0.05. For each repeated measures ANOVA, the partial eta squared (η^2^_p_) was presented as a measure of effect size. The Bonferroni post hoc test was used to examine whether any differences existed between the different conditions as the main ANOVA is significant. After the Bonferroni correction, the statistical threshold was adjusted to *p* < 0.016.

## Results

Table 1 summarizes the descriptive statistics and the results of the repeated measure ANOVA for the precision pinch performance, hand function, and sensory status under the three conditions of interest. A statistically significant difference was detected for the FR_peak_ parameter (F = 14.37, *p* < 0.001, η^2^_p_ = 0.507) and FP_peak_ (F = 7.295, *p* = .003, η^2^_p_ = 0.343) using the PHUA test, across all three conditions. Based on the post-hoc examination, the participants under the vibrotactile-enriched MBI condition had better capacity to modulate their pinch force according to the fluctuated load of the lifted object in terms of their dynamic pinch-lifting performance compared to the baseline and active MBI conditions (Table 1). However, there were no statistically significant differences for the precision pinch performance between the baseline and active MBI (*p* = 0.304 and *p* = 0.165, respectively, for FR_peak_ and FP_peak_).

**Table 1:** The descriptive statistics and the results of the repeated measure ANOVA used to determine the precision pinch performance, hand function, and sensory status under the three conditions

		Conditions			Difference across conditions		
		Baseline	Active MBI	Vibortactile-enriched MBI	F	*p*	η^2^_p_
PHUA	FP_peak_ (N)	13.19 ± 1.54	12.91 ± 1.15^‡^	12.40 ± 1.01^†‡^	7.295	.003	.343
	FR_peak_	2.98 ± .33	2.94 ± .30^‡^	2.76 ± .23^†‡^	14.370	< 0.001	.507
MTT (seconds)	Barognosis	3.18 ± .10	3.10 ± .09^‡^	2.81 ± .09^†‡^	19.126	< 0.001	.577
	Roughness differentiation	31.12 ± 6.40	28.56 ± 4.13^*‡^	27.49 ± 3.60^†‡^	15.036	< 0.001	.518
	Stereognosis	24.86 ± 2.65	23.46 ± 2.71^*^	22.40 ± 4.39^†^	9.057	0.001	.393
SWM test (gm)	Thumb	.181 ± .211	.156 ± .206	.130 ± .205^†^	4.389	.022	.239
	Index finger	.161 ± .170	.124 ± .153^*‡^	.083 ± .111^†‡^	6.373	.005	.313
	Little finger	.123 ± .143	.093 ± .109^‡^	.060 ± .073^†‡^	4.700	.017	.251
PPT	Pin insertion- DH	14.1 ± .4	15.0 ± .3^*^	15.2 ± .3^†^	8.454	0.001	.377
	Pin insertion- BH	11.3 ± 1.6	12.1 ± 1.2^*^	11.9 ± 1.4^†^	.11.932	< 0.001	.460
	Assembly	31.8 ± 5.9	35.6 ± 5.6^*^	34.6 ± 5.2^†^	18.458	< 0.001	.568

A repeated measure ANOVA was used to compare the effects of the different interventions. The level of significance was set a*t p* < 0.05. A post hoc test was used to examine whether any differences existed among the different conditions. After the Bonferroni correction, the statistical threshold was adjusted to *p* < 0.016

^*^: Significant difference between Baseline and Active MBI. ^†^: Significant difference between Baseline and Vibortactile-enriched MBI. ^‡^: Significant difference between Active MBI and Vibortactile-enriched MBI

*FR*_*peak*_ Force ratio (FP_peak_: FL_max_), *FP*_*peak*_ maximum pinch force during the lifting phase, *MTT* Manual tactile test, *SWM test* Semmes-Weinstein monofilament test, *PPT* Purdue Pegboard Test, *DH* Dominant hand, *BH* Both hands

In terms of sensory function, there was a statistically significant difference in the results of the MTT and SWM test for the participants across all three conditions (Table 1). The SWM results under the vibrotactile-enriched MBI condition was better than that under the baseline condition (*p* = 0.010, *p* = 0.001, and *p* = 0.004, respectively, for the thumb, index finger, and little finger). However, the SWM results under the active MBI condition revealed no statistically significant differences compared to the baseline condition (*p* = 0.069, *p* = 0.038 and *p* = 0.063, respectively, for the thumb, index finger, and little finger). The difference in the results of SWM between the vibrotactile-enriched MBI condition and active MBI condition was not statistically significant for the thumb, index finger and little finger (*p* > 0.016). Similar to the SWM results, the sensory assessment evaluation using three MTT subtests under the vibrotactile-enriched MBI condition revealed statistically better performance compared to the baseline condition (*p* < 0.001, *p* < 0.001 and *p* = 0.002, respectively, for barognosis, roughness differentiation, and stereognosis). In addition, compared to the active MBI, the participants spent relatively less time completing the barognosis (*p* < 0.001) and roughness differentiation (*p* = 0.014) subtests under the vibrotactile-enriched MBI condition. Different from the SWM results, better results were found in the roughness differentiation subtest (*p* = 0.006) and stereognosis (*p* = 0.001) following the active MBI as compared to the baseline condition; however, there was lack of statistical difference in the results for barognosis between the baseline and active MBI conditions (*p* = 0.077).

The results of the pin insertion subtests using the dominant hand, both hands, and the PPT assembly task revealed statistically significant differences across the three conditions (Table 1). The participants in both the active MBI and vibrotactile-enriched MBI conditions had faster performance in the three PPT subtests as compared to under the baseline condition.

## Discussion

The aim of the present study was to evaluate the differences in the effects on the sensorimotor control capacity of a hand across conditions including baseline, active MBI, and vibrotactile-enriched MBI conditions. A vibrotactile-enriched interfaces for MBI system suitable for sensorimotor control training was developed in this study. The findings partially supported the premise that the vibrotactile-enriched MBI would enhance the sensorimotor performance of a hand in healthy older adults. Participants in the vibrotactile-enriched MBI group exhibited better performance in a pinch-holding task that required online sensory feedback, as compared to those in the active MBI and the baseline groups. In addition, the results also showed that there were better effects of the vibrotactile-enriched MBI on improving sensory functions related to barognosis and roughness differentiation, as compared to those under the baseline and active MBI conditions. This result revealed an immediate effect of a single-session vibrotactile-enriched MBI on the sensorimotor performance of healthy older adults.

The FR_peak_ under the baseline condition in the current study was higher when compared with data obtained from young subjects [30]. A higher pinch-to-load force ratio, which indicated inaccurate pinch force modulation to the momentum-induced load changes [37], might have been due to the decline in the sensorimotor functioning of the hands. In the case of healthy participants, pinch force predictions and adjustments during execution of a functional task is an automatic motor response corresponding to the dynamics of arm movement [38]; however, recent evidence suggested that sensory deficits of a hand appear to remarkably decrease the capacity of momentary motor adjustment [39] because task-based sensorimotor processing depends on not only a feed-forward control mechanism but also on peripheral sensory events [40]. Since aging leads to slowing of sensorimotor functions [41], an intervention program involving the use of passive sensory stimulation drives plastic reorganizational changes in the sensorimotor cortex based on the Hebbian forms of plasticity [42], which promotes precision pinch performance in the hands of healthy seniors. The better performance in precision pinch performance when receiving the vibrotactile-enriched MBI intervention was supported by recent studies based on sensory augmentation systems used to explain the potential mechanisms of sensory restoration, sensorimotor integration, and substitution in motor control [43, 44]. That is, integration of afferent vibration signals in the form of haptic feedback in hand therapy contributes to enhancing hand performance [45].

In addition, the results of the SWM test and MTT test obtained under the baseline condition in the older adults examined in the present study revealed lower sensitivity in both the touch threshold [46] and discriminative sensory function [33]. The vibrotactile-enriched MBI had superior training effects on the results of the SWM test and the MTT barognosis subtest. Compared to the active MBI, the sensitivity related to both tactile and proprioceptive sensation increased through activation of sensory receptors during the transmission of vibrotactile stimulation to the finger tips, which enhanced sensory restoration in the participants receiving the vibrotactile-enriched MBI intervention. A recent report indicated that the vibrotactile component of the haptic feedback that occurs when playing a musical instrument leads to increased quality of hand perceptions [47]. Due to central mechanisms, vibrotactile stimulation on the fingertips results in not only activating cutaneous mechanoreceptors, but it also enhances the mechanical coupling between the contacted skin, tendons, and bones, which significantly improves the of touch-pressure threshold, as tested by Semmes-Weinstein monofilament [48] and the capacity of active force perception as measured when participants manipulate objects [49].

The active MBI in this study also presented some training effects on improving hand functions, which might concur with the findings of a previous review report regarding MST training used to enhance the motor functions of stroke survivors [50]. Active MBI provides visual and auditory information intended to guide the temporal and spatial organization of sequential motor responses based on rhythmic keyboard pressing and enhances the coordinated actions of the hand [51]. Furthermore, the predictability of motor sequences serves as a facilitative factor for motor control based on a cognitive processing mechanism that occurs during the preparation and execution of movements such as keyboard pressing during active MBI [52]. Therefore, music-based paradigms have been suggested to be an effective strategy for motor learning and rehabilitation. Also, the results of the roughness differentiation and stereognosis after the active MBI revealed statistically significant changes compared to the baseline condition in the current study, which might have been correlated with the enrichment of the sensorimotor network through functional motor training in a multisensory environment [53].

This may be the first study examining the effects of an intervention using a music-based intervention with multiple sensory feedback on sensorimotor performance of the hands of healthy senior participants. This strengths of the current pilot study include the potential training device innovation and comprehension of its feasibility for future clinical application. A novel vibrotactile-enriched MBI system provided synchronized auditory, visual and vibrotactile stimuli related to music characteristics of melody and rhythm was proposed in this study. The present study established a vibrotactile-enriched MBI system acting as a sensory augmentation approach to dealing with motor control capabilities in the hand of an aging brain. It is noteworthy that better training results in precision pinch performance, hand function, and sensory function of healthy older adults have been obtained for participants even when only receiving the vibrotactile-enriched MBI intervention once comparing with receiving the active MBI. Also, both the vibrotactile-enriched MBI and active MBI interventions had beneficial effects on hand performance and sensory function compared to the baseline condition. The obtained results support the feasibility of clinical application of a sensory augmented MBI. However, several limitations exist in the present study. First of all, the protocol only provided immediate outcome measures, which make it impossible to clearly understand the delayed effects on the sensorimotor performance that occurred in both the vibrotactile-enriched MBI and active MBI interventions. The second limitation was lack of assessment done at the end of the washout period to ensure a true washout effect of the previous treatment. The third limitation was that the design of current study was only a single session intervention, which did not permit us to observe effects of a longer training period of music-supported therapy on the sensorimotor performance of hands. In spite of these limitations, the results indeed have merit related to supporting future studies investigating the impacts of vibrotactile-enriched MBI and active MBI on hand sensorimotor performance or sensory functioning in participants with marked sensorimotor deficits. However, the questionnaire used to measure the response regarding sensory information perceiving and reality of key pressing for MBI experiences in the two conditions needs be conducted further.

## Conclusions

In summary, the pilot study supports the feasibility of using vibrotactile-enriched MBI for ameliorating sensorimotor performance in the hands of healthy older adults. Future studies are needed in order to optimize the training protocol of music-supported therapy by examining the effects of intervention intensity or time periods required for executing vibrotactile-enriched MBI and active MBI training in individuals with age-related sensorimotor deficits or neurological impairments.

## Data Availability

Not Applicable.

## Abbreviations

MBI: Music-based intervention
PHUA: Pinch-holding-up activity
FP_peak_: Peak pinch force
FL_max_: Maximum load force
FR_peak_: Ratio between peak pinch force and maximum load force
MTT: Manual tactile test
SWM: Semmes-Weinstein monofilament
PPT: Purdue Pegboard Test
SPSS: Statistical Package for Social Sciences
ANOVA: Analysis of variance
